# Disruption of a Regulatory Network Consisting of Neutrophils and Platelets Fosters Persisting Inflammation in Rheumatic Diseases

**DOI:** 10.3389/fimmu.2016.00182

**Published:** 2016-05-19

**Authors:** Norma Maugeri, Patrizia Rovere-Querini, Angelo A. Manfredi

**Affiliations:** ^1^San Raffaele Scientific Institute, Università Vita Salute San Raffaele, Milano, Italy

**Keywords:** platelets, neutrophils, HMGB1, inflammation, rheumatic diseases

## Abstract

A network of cellular interactions that involve blood leukocytes and platelets maintains vessel homeostasis. It plays a critical role in the response to invading microbes by recruiting intravascular immunity and through the generation of neutrophil extracellular traps (NETs) and immunothrombosis. Moreover, it enables immune cells to respond to remote chemoattractants by crossing the endothelial barrier and reaching sites of infection. Once the network operating under physiological conditions is disrupted, the reciprocal activation of cells in the blood and the vessel walls determines the vascular remodeling *via* inflammatory signals delivered to stem/progenitor cells. A deregulated leukocyte/mural cell interaction is an early critical event in the natural history of systemic inflammation. Despite intense efforts, the signals that initiate and sustain the immune-mediated vessel injury, or those that enforce the often-prolonged phases of clinical quiescence in patients with vasculitis, have only been partially elucidated. Here, we discuss recent evidence that implicates the prototypic damage-associated molecular pattern/alarmin, the high mobility group box 1 (HMGB1) protein in systemic vasculitis and in the vascular inflammation associated with systemic sclerosis. HMGB1 could represent a player in the pathogenesis of rheumatic diseases and an attractive target for molecular interventions.

## Neutrophils, Platelets, and Vascular Inflammation

Neutrophils are terminal cells with a relatively short half-life in the circulation. They are effective as a first barrier toward various invading noxae. To carry out this function, neutrophils cross the vessel wall and migrate to the inflamed/injured tissues. This step requires the recognition of P-selectin on the activated endothelium and the asymmetric polarization of the leukocyte β2 integrins that generates the unidirectional movement associated with the infiltration of the surrounding perivascular tissues. These events influence the immune function of transmigrating leukocytes, thus contributing to the overall outcome of the inflammatory response: effective resolution versus persistence of vascular inflammation, healing of the injured vessel wall versus active remodeling, intimal hyperplasia, or aneurism formation. Conversely, transmigrating neutrophils both damage and activate endothelial cells, enforcing a self-sustaining positive feedback loop that contributes for example in patients with systemic small-vessel vasculitis or with systemic sclerosis (SSc) to vascular remodeling and inflammation (1–4).

At sites of infection, neutrophils dispose of invading microorganisms. This action depends partially on the engulfment into a phagosome upon reorganization of the actin-based cytoskeleton and on the activation of the NADPH oxidase system with generation of reactive oxygen species (ROS) (5, 6). The oxygen species combined with the granules microbicidal moieties released into the phagolysosome limits the pathogen viability (6–8). Non-phagocytic neutrophils’ microbicide mechanisms have also been described, which involve the release of decondensed chromatin threads in the extracellular space. This phenomenon is referred to as neutrophil extracellular traps (NETs) generation (8–11). Neutrophils preferentially generate NETs when they fail to engulf the pathogen because they are immobilized, tightly adherent to a substrate, or near to apoptosis (8, 12–15). Primary granules fuse with the nuclear membrane, causing the formation of myeloperoxidase–DNA and elastase–DNA complexes (14), while the physicochemical properties of the chromatin change dramatically upon the citrullination of histones by the peptidylarginine deiminase 4 (PAD4) enzyme (16, 17). Both granule redistribution and PAD4-mediated histone citrullination are required for NET generation. During experimental sepsis, NETs play a role in bacterial trapping ensnaring circulating bacteria and restricting their dissemination to distant organs (18).

Neutrophils and platelets colocalize at sites of vascular injury, hemorrhage, and thrombosis. In these conditions, various inflammatory and thrombogenic signals are integrated, resulting in the productive interaction between platelets and leukocytes, yielding the formation of aggregates (15, 19, 20). Neutrophils/platelets heterotypic aggregates depend on platelet P-selectin, are endowed with inflammatory and thrombogenic actions, and represent a shared feature of acute cardiovascular diseases and of systemic inflammatory, neoplastic, and autoimmune diseases (21).

**Figure 1:**
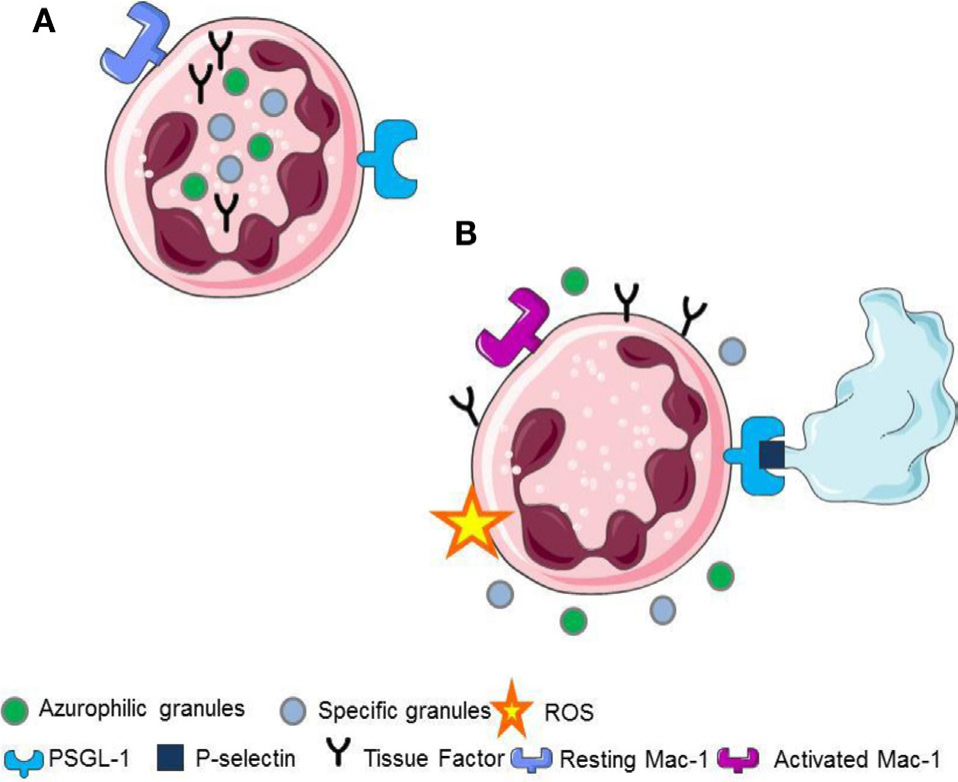
**(A)** Resting neutrophils. **(B)** P-selectin recognizes PSGL1 promoting the generation of ROS, the activation of Mac-1 on neutrophils, the release of pentraxin 3 from the neutrophil-specific (secondary) granules, the release of myeloperoxidase from the neutrophil azurophilc (primary) granules and the surface expression, and the novo synthesis of tissue factor in neutrophils.

Upon platelet adhesion to damaged vessel walls, P-selectin expressed on their surface facilitates the leukocyte recruitment at the site of vascular injury. Signals activated downstream the recognition of platelet P-selectin promote the generation of ROS (5, 22), the activation of β2 integrins (2, 7, 15, 23, 24), the release of pentraxin 3 from the neutrophil specific (secondary) granules (25), the release of myeloperoxidase from the azurophilic (primary) granules (6, 25, 26), and the *de novo* synthesis and the surface expression of leukocyte tissue factor (1, 2, 21) (Figure 1).

Additionally, activated platelets release soluble inflammatory signals, including IL-1β, PDGF, and the prototypic endogenous immune adjuvant, the high mobility group box 1 (HMGB1) protein (5, 27, 28). Finally, the inflammatory action of platelets is amplified and sustained by the release of bioactive microparticles (5, 20, 27, 29, 30). Microparticles comprise small vesicles (usually ranging from 0.05 to 1 μm) shed from activated or dying cells as a consequence of the disruption of the pathway actively maintaining the asymmetry between the phospholipid layers of the plasma membrane. Most microparticles in the blood derive from platelets (30). Platelet-derived microparticles participate in blood coagulation and actively contribute to the inflammatory action of platelets (30). The array of signals expressed, generated, or released by platelets upon activation acts mostly locally influencing the microenvironment. However, these signals possibly influence the leukocyte function in the circulation, in particular in patients with systemic vasculopathy (2, 31).

Of importance, the dangerous connection between neutrophils and platelets can have different outcomes depending on the context. These outcomes include the phagocytic removal of platelets in physiological conditions (6). Upon inflammatory conditions, adherent neutrophils recognizing activated platelets are committed to NET generation (12, 20, 32).

## NETs Contribute to Propagate Vascular Injury and Autoimmunity

**Figure 2:**
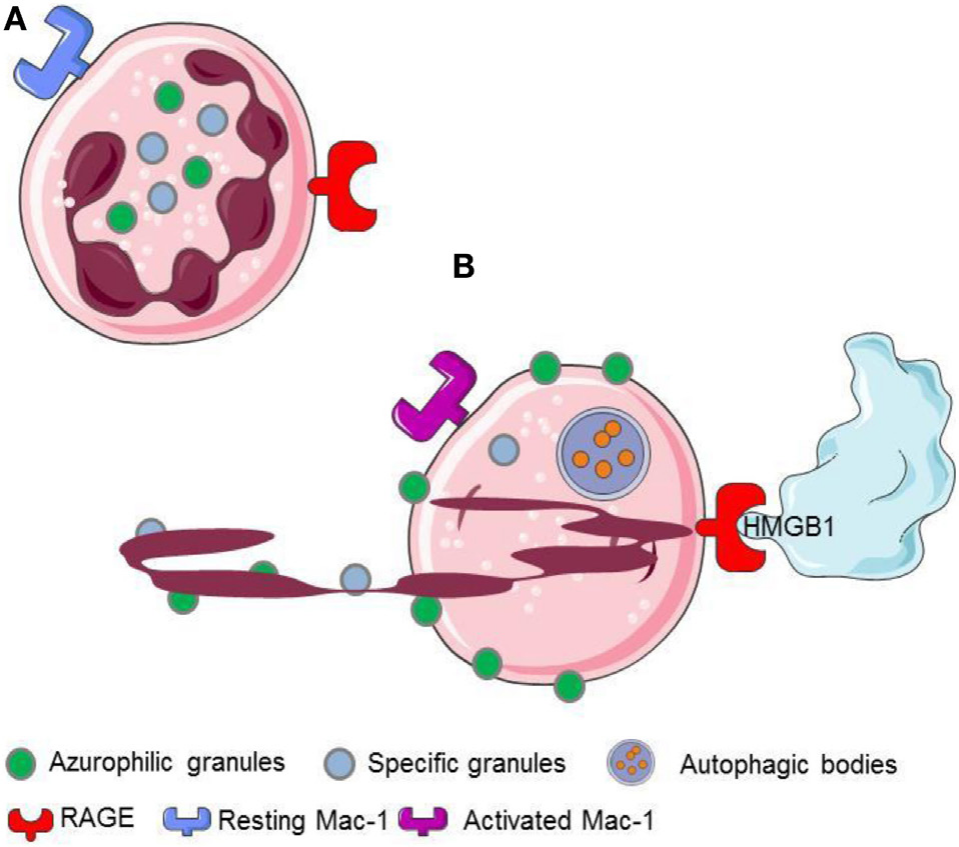
**HMGB1 released or expressed by activated platelets recognizes RAGE expressed on neutrophils**. **(A)** Resting neutrophils express non activated Mac-1 and RAGE on their surface. **(B)** HMGB1 expreesed by activated platelets induces neutrophils to initiale autophagy, promotes the redistribution of neutrophil granules, induces the transactivation of Mac-1 and elicits the NETs formation.

**Figure 3:**
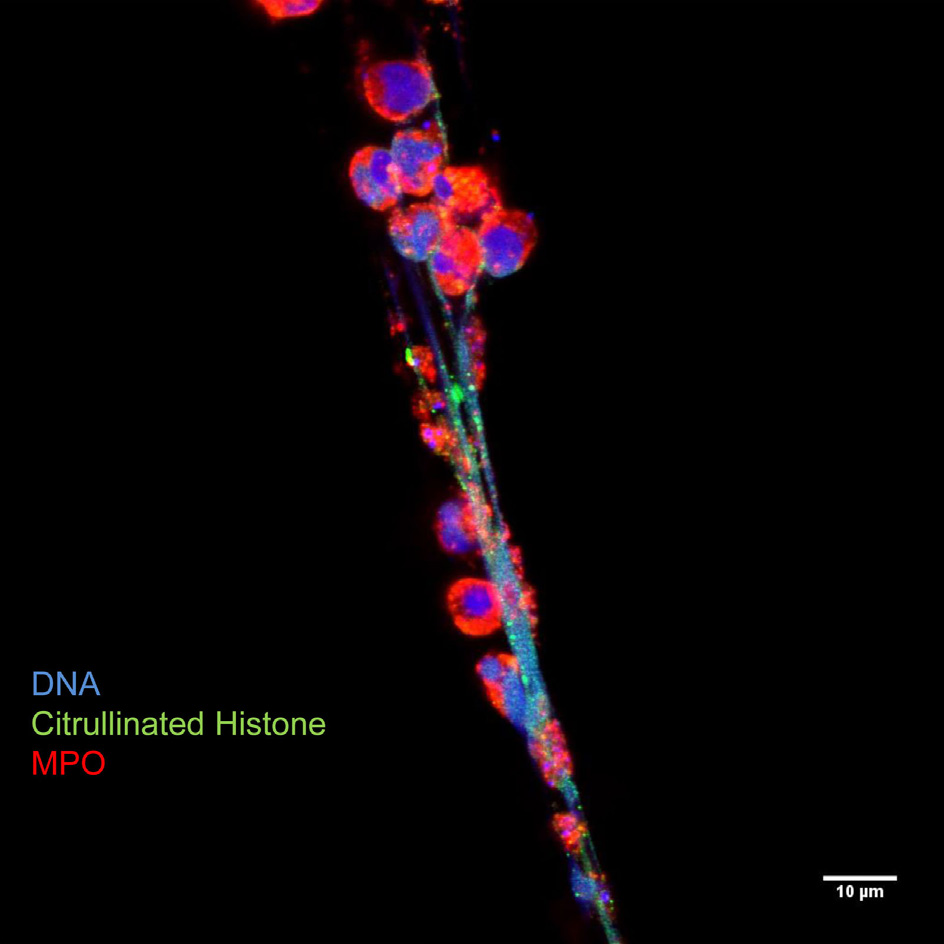
**Neutrophil extracellular traps are characterized by decondensed latices of DNA with citrullinated histones decorated with neutrophil granules proteins, such as myeloperoxidase**. Originally published by Maugeri et al. (20).

Neutrophil extracellular traps generation comprises a physiological response of living neutrophils to various stimuli present in a specific environmental context (11, 12, 20, 33) or a form of cell death that is morphologically distinct from apoptosis (34, 35). The mechanisms regulating the type of NET formation seem to depend on the triggering stimuli and on the context of stimulation. They comprise (i) the production of ROS and the induction of autophagy, (ii) the fusion of primary granules with nuclear membrane, (iii) the interaction of elastase and MPO with the DNA, (iv) the citrullination of histones, the chromatin decondensation, and, finally, (v) the nuclear envelope and, eventually, the cell membrane integrity disruption (10, 34, 36) (Figures 2 and 3).

The ensuing prolonged exposure of neutrophil microbicidal proteins as well as of citrullinated histones in the extracellular environment could initiate autoimmunity (4, 10, 37, 38). NETs are cleared *via* a mechanism involving DNAses and the first component of the classical pathway of complement activation, C1q. As a consequence, the endocytotic/phagocytotic function of scavenger macrophages limits NETs inflammatory and potentially vessel-injuring properties (39, 40). Moreover, persistence of NETs in the tissue might prompt fibrosis *via* induction of myofibroblasts (41).

In contrast, defective clearance of NETs and activation of the alternative pathway of complement activation might be associated with persisting tissue damage and to autoimmunity (10, 42–46). Accumulation in the plasma of byproducts of NET generation/catabolism (such as complexes of DNA-MPO or soluble DNA with citrullinated histones), higher capacity of *in vitro* NETs generation, and an impaired capacity of NETs degradation were observed in patients with systemic lupus erythematosus (SLE), small vessel vasculitis, rheumatoid arthritis, and psoriasis (47), while the association of biologically active moieties, such as tissue factor, TNFα and IL-1β, with NETs might influence their action in the microenvironment (48, 49).

The formation of NETs *in vivo* seems to be directly associated with SLE. Accordingly with this hypothesis, in an animal model of lupus, the effective inhibition of PAD4 results both in a reduction of NET formation and in altered circulating autoantibody profiles, restored complement levels, and reduced glomerular IgG deposition (37, 38, 50).

## Endogenous Mechanisms Maintaining Vascular Inflammation: A Role for HMGB1

High mobility group box 1 has been named after its ability to quickly migrate in polyacrylamide and triton–urea gels, a feature that depends on a high-content of charged amino acid residues. HMGB1 is located on the 13q12 human chromosome. The gene comprises six exons that encode for a 215-amino acid polypeptide, with an apparent molecular mass of 25 kDa. HMGB1 proteins from mammals are nearly identical, indicating that each residue is under selective pressure. In general, the cell type and state of activation influence levels and localization, and more differentiated cells display a lower protein content. HMGB1 consists of a long acidic carboxyterminal region (the “acidic tail”) and of two positively charged domains, referred to as “box A” and “box B,” that bind to DNA and contain nucleus localization signals. HMGB1 is mostly located in the nucleus of most living cells where it bends DNA, thus facilitating the assembly of proteins, including transcription factors, on their targets. HMGB1 moves constantly from the nucleus to the cytoplasm (51, 52). In response to stress, senescence, or inflammatory signals, HMGB1 is hyperacetylated at two sites in nuclear localization, and this isoform accumulates in the cytoplasm (53). HMGB1 in the cytoplasm promotes autophagy, by which cells recycle internal constituents so as to generate ATP and promote survival under conditions of environmental stress (51).

The interaction with the chromatin in living cells is transient, and HMGB1 plays relevant biological functions in the cytosol, where it behaves as a potent inductor of autophagy (54). Cell death *via* an unscheduled accidental pathway, which associates with the disruption of membrane compartmentalization, results in the redistribution of the molecule at extracellular sites, where HMGB1 behaves as a potent inflammatory signal (51, 55). Most activated cells also mobilize HMGB1. Monocytes, macrophages, and immature myeloid and plasmacytoid dendritic cells (DCs) secrete HMGB1 in response to primary inflammatory signals. Anucleated platelets also contain and upon activation release substantial amount of HMGB1, either as a soluble moiety or associated with microparticles (5, 20, 27, 28). HMGB1 released or expressed by activated platelets commits neutrophils to autophagy (20), promotes the redistribution of neutrophil granules (5), induces the Mac-1 transactivation (5), and elicits the NETs formation (20, 56, 57) (Figure 2).

Posttranslational modifications, such acetylation, phosphorylation, methylation, and oxidation/reduction (58–61), and the interaction with other bioactive molecules, including LPS or chemokines (62, 63), CXCL12 in particular (64, 65), all influence the function of extracellular HMGB1 (5, 58, 66). The redox status of the three cysteine residues of the molecule (C23, C45, and C106) apparently dictates the sequential events of leukocyte recruitment, activation, and resolution of inflammation (59). The characteristics of HMGB1 biology, including its association with various events important in the natural history of vasculitis, such as necrosis, granuloma formation, and leukocytes survival and activation, as well as its ability to regulate inflammation and tissue repair and remodeling, make the protein a candidate player in this family of diseases.

Acute vascular inflammation has a well-characterized homeostatic role. Defects in the program result in self-sustaining vascular inflammatory diseases, referred to as vasculitis. Indeed, an unrelenting inflammatory process mostly restricted to the vessel wall characterizes large vessel vasculitis [Takayasu arteritis, giant-cell arteritis (GCA)] (3, 67). The productive interaction between activated adventitial DCs and T cells is an early and crucial event. The local production of T cells cytokines eventually results in IFNγ-mediated activation of macrophages and in the formation of giant cells at the intima-media junction. Giant cells and activated macrophages produce growth factors (such as vascular endothelial growth factor and platelet-derived growth factors), which sustain intimal hyperplasia and contribute to subsequent end organ ischemia (68). Circulating blood cells are also activated and might contribute to the clinical picture. For example, thrombocytosis is frequent in GCA patients (69, 70), and aspirin protects patients from cranial ischemic complications (1, 71–74). Aspirin-resistant events are, however, quite frequent, and platelet count does not identify patients at higher risk of severe ischemic events (74–76).

Blood cells of GCA patients express tissue factor, a key molecule in thrombin formation downstream activation of Factor VII, and display a greater fraction of platelets expressing P-selectin, which is associated with a procoagulant state (1, 77). Specific clinical features or the extent of biomarkers of systemic inflammation, which, however, may fail to reveal the extent of ongoing smoldering vascular inflammation (78), do not apparently influence these features (3). Despite extensive investigations, markers reflecting not exclusively inflammation but the extent of the process taking place in the affected vessels have proved elusive, with the possible exception of the soluble pattern recognition receptor PTX3, which is produced in peripheral tissues in response to signals of injury by innate immune cells, such as DCs and macrophages (79, 80).

Dendritic cells and macrophages are a critical source of HMGB1, which shapes their functional polarization and migratory properties (81–85). While PTX3 plasmatic levels seem to be associated with the entity of the disease (79, 86, 87), concentrations of HMGB1 in the blood are not an effective biomarker of large vessel vasculitis (88). Indeed, patients with Takayasu’s arteritis and GCA present similar serum HMGB1 levels compared with healthy controls and seem unrelated to disease activity (88).

However, it should be considered that several posttranslation modifications influence the bioactivity of the molecule. Specifically environmental conditions, such as the redox status, influence HMGB1 inflammatory action, causing the shift from a moiety that mostly causes leukocyte recruitment or to a signal that elicits the secretion of inflammatory cytokines (53), see above. As such, the total concentrations of the molecule in the blood might not reflect the actual fraction of the bioactive molecule (5, 53, 59). When potent inflammatory molecules are released in the environment, inhibitors are often physiologically generated, like it occurs for the primary inflammatory cytokines, TNFα and IL-1β. The identification of putative HMGB1 inhibitors requires further study. The development of analytical techniques to discriminate among the various forms of HMGB1 might allow to dissect the actual HMGB1 involvement in the various facets of vascular inflammation: effective repair of injured vessels, angiogenesis, persistent inflammation with extensive remodeling, aneurysm formation, development of atherosclerotic lesions, complications associated with their disruption, etc. (89).

Leukocytoclasia (i.e., the presence of of uncleared leukocyte debris within and around the vessel wall), small-vessel thrombosis, necrosis, and hemorrhage in target organs (mainly the skin, the kidneys, and the airways) are hallmarks of small-vessel vasculitis. Immune complexes play a major role in eliciting vascular inflammation during some small-vessel vasculitis (IgA vasculitis or cryoglobulinemia, for example), and immunoglobulin and complement deposition at the site of vascular injury accompanies in these patients’ leukocytoclasia. In contrast, a “pauci-immune” inflammation, without local immunoglobulin or complement deposition, characterizes vasculitis syndromes associated with antineutrophil cytoplasmic antibodies (ANCA-associated vasculitis). Elevated levels of plasmatic HMGB1 have been found in patients with small-vessel vasculitis, including IgA vasculitis, Kawasaki’s disease, and ANCA-associated vasculitis (90–93). The concentration of plasmatic HMGB1 is elevated in the active phase of systemic vasculitis, and the concentration of HMGB1 is higher in patients with granulomatosis with polyangiitis with a predominantly granulomatous disease (94). In contrast, conventional markers of inflammation or the validated disease activity score, BVAS, fail to discriminate between the two groups of patients (94). The result well fits the preferential expression of the HMGB1 in the granulomatous tissue (94) and suggests that systemic levels might actually reflect local *in situ* production. HMGB1 levels are also been described to be higher in patients with active renal involvement, a threatening manifestation of the disease (90). Levels of HMGB1 are still elevated in patients with a quiescent nephritis, possibly indicating a persistent low-grade inflammation that persists in the subclinical phases of the disease (90). Urinary levels of HMGB1 represent a robust biomarker of active glomerulonephritis in patients with ANCA-associated vasculitis (42, 95). Actually, urinary HMGB1 might represent a more solid biomarker of kidney involvement in ANCA-associated vasculitis than serum HMGB1 (96).

The preferential involvement of HMGB1 in ANCA-associated vasculitis might be related to its ability to regulate the activation state and function of neutrophils, which are the key cells in the pathogenesis of these diseases. Of interest, HMGB1 could contribute as inflammatory priming of neutrophils in circulation, inducing translocation of ANCA antigens at cell membrane, providing the substrate of antigen–antibody interactions (97).

**Figure 4:**
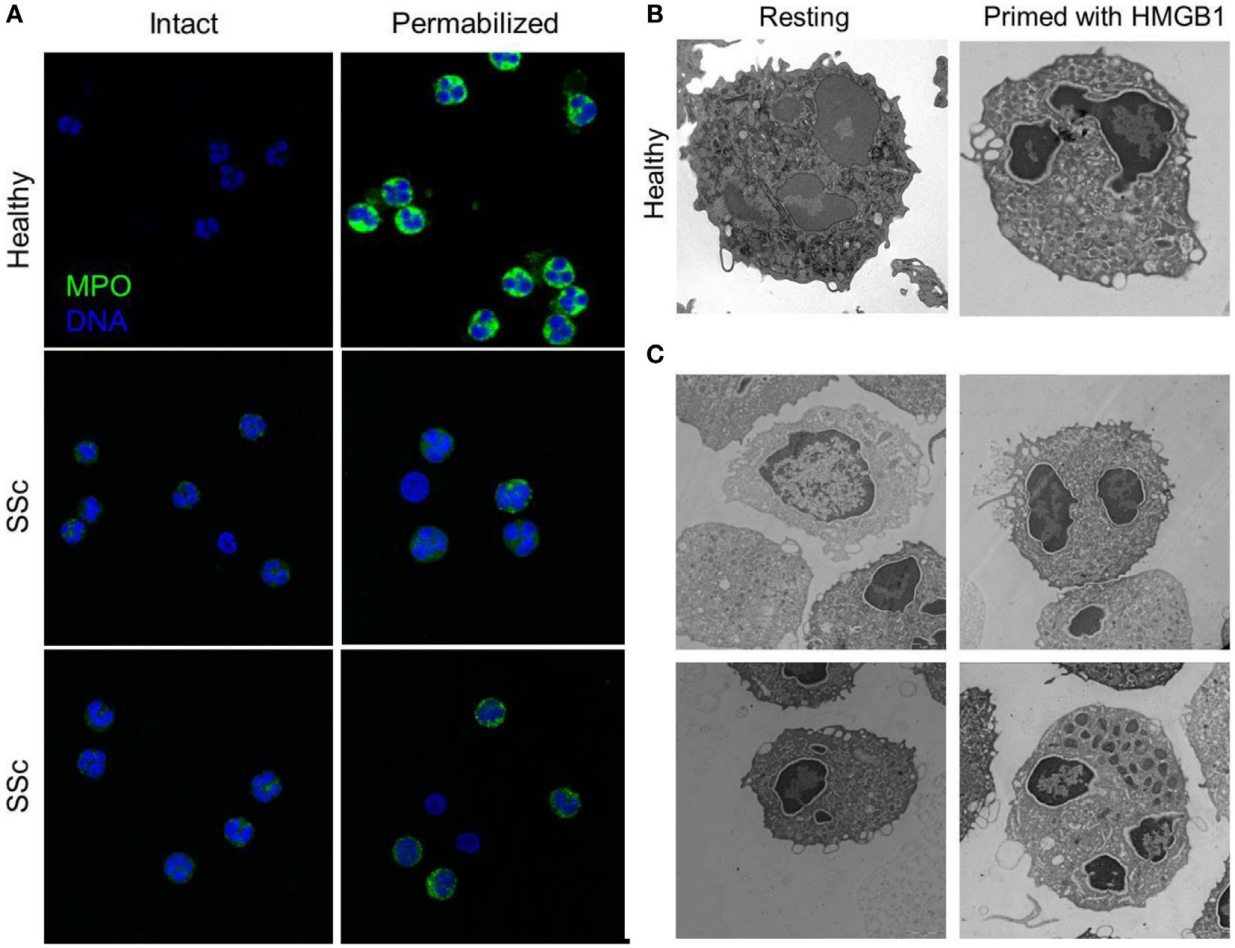
**Peripheral granules distribution characterized neutrophils of patients with SSc**. **(A)** The expression of MPO (green) in the blood neutrophils of patients with SSc and of matched controls has been analyzed by confocal microscopy before and after permeabilization of the plasma membrane, to allow the access of the mAb. MPO intracellular expression is substantially lower in SSc patients and appears to cluster at the plasma membrane of intact, non permeabilized neutrophils. **(B,C)** Representative images by electron microscopy of neutrophils from a healthy control, untreated or treated with HMGB1 **(B)** or of untreated neutrophils from four SSc patients **(C)** showing the extensive remodeling of intracellular granules, most of which acquire a pericellular distribution, characterizes SSc neutrophils and healthy neutrophils treated with HMGB1. Images originally published by Maugeri et al. (5).

The recognition of extracellular HMGB1 dramatically influences several characteristics of neutrophils, a key population in ANCA-associated small-vessel vasculitis. It induces a swift redistribution of intracellular vesicles, an event that might be associated with the ability to activate neutrophil autophagy (5) through the putative HMGB1 receptor, the receptor for advanced glycation endproducts (RAGE) (Figures 2 and 4).

The redistribution of the granule in response to primary inflammatory stimuli allows the exposure of ANCA antigens (namely, myeloperoxidase and proteinase 3) on the neutrophil plasma membrane, where they become accessible for interaction with the ANCA autoantibodies because of a preferential location at lipid rafts that also contain β2 integrins, signalling molecules, cross-linked Fcgamma receptors, and NADPH oxidase. ANCA, in turn, amplify the activation of the neutrophil, which is transmigrating, favouring a vigorous and untimely response, with oxidative burst and premature degranulation (97, 98). HMGB1 might, thus, act on neutrophils favouring the exposure of ANCA antigens and facilitating the further neutrophil activation caused by the antigen recognition by ANCAs. Of importance, HMGB1 has been recently shown to potentiate the NETs formation induced in the presence of ANCAs (99).

## HMGB1 and Diabetic Vasculopathy

High mobility group box 1 elevation appears as a relatively shared feature in patients with an inflammatory vascular involvement. This applies not only to other primary vasculitides (91, 100, 101) but also to other systemic diseases characterized by extensive inflammatory vessel involvement (102). Diabetes mellitus represents a privileged scenario for the study of the role in vascular inflammation of HMGB1 and of the RAGE receptor. The systemic HMGB1 concentration is consistently elevated in diabetic patients and in animal models of the disease (103, 104). HMGB1 might contribute to the accelerated atherosclerosis, which is a hallmark of diabetes mellitus (105–107). Hyperglycemia is an effective stimulus leading the release of HMGB1, which in turn might play a role in the failure of tolerance in diabetes mellitus type 1 (108, 109) and in the early rejection of transplanted islets (110, 111). Furthermore, glycated albumin is recognized by RAGE inducing the neutrophil activation and release of NETs (20).

## HMGB1 and Systemic Sclerosis

Systemic sclerosis is an immune-mediated multisystem disease, characterized by a diffuse obliterative microvasculopathy and by fibrosis of the skin and of visceral organs. The abnormal generation of ROS observed in patients with SSc contributes fostering autoimmunity, fibrosis, and vascular inflammation. Recently, the presence of an increased concentration of platelet-derived microparticles (PDμP) bearing HMGB1, P-selectin expressing platelets (5, 27), the redistribution of the content of primary granules, and the transactivation of β2 integrins leukocytes was observed in blood cells of SSc patients (Figure 4). P-selectin (purified or expressed on activated platelets) induces the ROS generation by neutrophils, which in turn cause the oxidation of the HMGB1 expressed by PDμP. Oxidation amplifies the ability of HMGB1-expressing PDμP to activate neutrophils, favoring the redistribution of molecules present in the neutrophil primary granules to the plasma membrane and the transactivation of β2 integrins. Leukocyte activation caused by oxidized extracellular HMGB1 abates in the presence of inhibitors of HMGB1 or of catalase, which catalyzes the dismutation of hydrogen peroxide into water and molecular oxygen. Neutrophils from healthy donors challenged with HMGB1-expressing PDμP purified from SSc patients, but not those purified from control subjects, reproduce the phenotype of neutrophils of SSc patients, whereas HMGB1 inhibitors reverse the effects of microparticles (5, 27). These results suggest that HMGB1 might represent a crucial signal in the cross talk between platelets and leukocytes in SSs, thus sustaining microvascular inflammation. Its ability to promote epithelial and endothelial to mesechimal transition might further link vascular inflammation to the other prominent feature of SSc, fibrosis (112).

## HMGB1: A Player in Angiogenesis and Thrombosis

Platelet-derived HMGB1 appears as a crucial signal in the cross talk between platelets and leukocytes with potent and specific effects in the regulation of the ability of neutrophils to generate NETs and to activate the autophagic flux (20).

Neutrophil extracellular traps have a well-characterized role in thrombosis, and HMGB1 appears as a player in coronary thrombi formation in patients with acute myocardial infarction (20). A primary role of platelet-derived HMGB1 in thrombosis induction has been confirmed in an elegant genetic model relying on transgenic mice in which the molecule has been specifically ablated (56).

Mechanical or immune-mediated injury of vessels and ischemia/reperfusion cause HMGB1 release (113–117). HMGB1 blockade substantially improves the clinical outcome in several such models, indicating that HMGB1 broadcasts news of ongoing tissue injury and is involved in the ensuing inflammatory response. HMGB1 acts on virtually all cell populations involved in vascular inflammation. It is produced by injured endothelial cells and attracts endothelial cell precursors, which favor neovascularization. HMGB1 overexpression activates a pro-angiogenic program in endothelial cells, mediated *via* the increased activity of matrix metalloproteinases, of intregrin receptors, and the activation of the NF-κB pathway (118).

Thus, HMGB1 might represent a crucial event to switch the homeostatic inflammatory response to acute vessel injury to self-sustaining vasculitis (3). DCs play a critical role in the establishment of small vessel vasculitis (85, 119–121) and in the vessel wall inflammation, which characterize large vessel vasculitis (122, 123). HMGB1 prompts its own autocrine/paracrine release, enforcing a vicious circle, which is further amplified by other cytokines known to elicit HMGB1 release, including IL-1β and TNF-α (55, 82, 83). Finally, the ability of HMGB1 to prompt angiogenesis [see above and Ref. (124)] and to attract vessel-associated stem cells (125) might contribute to intimal hyperplasia/neo-angiogenesis, typical of vessel remodeling during large artery vasculitis.

The events that are implicated in this amplificatory loop associated are not completely characterized. For example, pericellular myeloperoxidase distribution could directly implement the HMGB1/RAGE pathway (5), since the myeloperoxidase system of human neutrophils generates *N*″-(carboxymethyl) lysine, a highly reactive advanced glycated end product and RAGE–ligand, at sites of inflammation (126).

Thrombosis is a common and often underestimated complication of ANCA-associated vasculitis (127, 128). Thrombosis occurs as a clinically apparent event and can often in active lesions biopsies be identified at the microscopic levels (129). A study comparing platelet and neutrophil activation of patients with acute coronary syndromes and autoimmune diseases demonstrated that the average of neutrophil myeloperoxidase content in patients with ANCA-associated vasculitis is similar to the one observed in patients with no segment T elevation myocardial infarction or unstable angina, while the fraction of neutrophil expressing the activated isoform of Mac-1 and platelets expressing P-selectin is similar to all acute coronary syndromes studied (26). Enhanced concentrations in the blood of markers of platelet activation, soluble P-selectin, and CD154 directly correlate with disease activity in large cohort of patients with granulomatosis with polyangiitis (130) [see also Ref. (26)]. P-selectin and CD154 are both involved in the physical interaction and mutual activation of platelets and neutrophils. Their increased turnover in patients with ANCA-associated vasculitis might reflect the link between vascular damage and thrombosis (3). Disrupted endothelial layers recruit and activate platelets, with ensuing activation of the coagulation system cascade. Moreover, platelet P-selectin expression compensates for the lack of endothelial P-selectin, making neutrophil rolling and extravasation possible [discussed in Ref. (7, 131)]. Neutrophil activation implies the release of protease, which contribute to platelet P-selectin and CD154 cleavage, whose circulating levels consequently increase.

High mobility group box 1 acts as a prototypic agonist for a variety of innate receptors, including RAGE, TLR2, TLR4, TLR9, TREM1, and Mac-1. *Via* these receptors, HMGB1 in pathological conditions perturbs vessel integrity and contributes to maintain the vicious cycle by which the inflamed endothelium increases the adhesion and the transmigration of leukocytes, and leukocytes, in turn, sustain the activation of endothelial cells, eventually leading to cell death and to the activation of programs that might sustain further vessel injury and thrombosis, such as the generation of NETs (132–134).

## Endothelial Response to HMGB1

Endothelial cells express are exquisitely sensitive to extracellular HMGB1. They express an array of HMGB1 receptors, which comprises RAGE, TLR2, TLR4, TREM1, proteoglycans, and thrombomodulin. The outcome of HMGB1 recognition by endothelial cells dramatically differs depending on which receptors are activated in the various conditions (135). A net activatory effects apparently ensues TLR2 or TLR4 activation, as assessed by the upregulation of adhesion molecules, by the production of cytokines, by the increased vascular permeability, by the activation of the coagulation system, resulting in certain conditions in microvascular thrombosis. HMGB1 not only *per se* activates endothelial cells (136, 137) but also behaves as a general adaptor of the ability of the endothelia to response to various sterile noxious signals. For example, uric acid recruits a complex series of events, including the enhanced expression of the HMGB1-mRNA, the acetylation of HMGB1, its translocation to the cytoplasm, and eventual release. In turn, HMGB1 recognition activates a positive feedback loop causing further HMGB1 expression and release (117).

The outcome of HMGB1 recognition by endothelial cells is finely regulated: this is expected, given the abundance of the molecule and the relatively easy access to the extracellular environment in case of cell activation or death. This might imply the recruitment of pathways that protect the host against the inflammatory action of endogenous components, in particular the CD24–Siglec pathway (138, 139) or the thrombomodulin-dependent pathway. Thrombomodulin is an evolutionary conserved glycosylated type I transmembrane protein with multiple functional domains, which is expressed by endothelial cells, endowed with anticoagulant actions. The thrombomudulin/thrombin complex activates protein C and in the presence of protein S interferes with factors VIIIa and Va and quenches thrombin generation (140). Thrombomulin ensures vessel homeostasis under stress and a rapid and localized inflammatory response to injury. Indeed, besides thrombin, thrombomodulin interacts *via* independent domains with various other molecules, including fibrinolysis inhibitors, complement components, and HMGB1. The interaction of HMGB1 with the lectin-like domain of thrombomodulin attenuates inflammation. This might be due to interactions with intermediary proteins that quench the endothelial cell activation (140, 141). Moreover, the binding to the lectin-like domain of thrombomodulin might limit HMGB1 binding to RAGE, thus impairing NF-κB activation (141). Thrombomodulin also enhances thrombin-mediated proteolytic degradation of HMGB1, reducing its pro-inflammatory activity (142). Since HMGB1 has been linked to the pathogenesis and/or progression of a large range of clinical disorders characterized by endothelial dysfunction, including sepsis and autoimmune diseases, the identification of thrombomodulin as a natural inhibitor of HMGB1 is of clinical importance (140, 142, 143) (Table 1).

**Table 1:** **Comparison of some features of vascular inflammation in sepsis and ANCA-associated small-vessel systemic vasculitis**.

	Sepsis	Vasculitis
Platelet count	Frequently low	Normal
Neutrophil count	High	Normal
Platelet activation	Yes	Yes
Neutrophil activation	Yes	Yes
Apoptotic neutrophils in circulation	Yes	No
Endothelial activation	Yes	Yes
Plasma thrombomodulin level	Low	Normal or high
HMGB1	High	High
NETs	High	Not documented in all types of vasculitis

During acute phases of vessel inflammation, thrombomodulin expression on the endothelial surface decreases because of at least two mechanisms: (i) internalization by endocytosis (144) or (ii) cleavage by enzymes like neutrophil elastase or cathepsin G (145, 146). Indeed, high levels of plasma thrombomodulin charaterize patients with systemic vasculitis [e.g., see Ref. (147–149)]. Whether the soluble cleaved thrombomodulin maintains the ability to bind to HMGB1 and whether the complex retains biological activities remain to be established.

Several other mechanisms possibly contribute to quench the inflammatory and thrombogenic actions of HMGB1 in the blood. For example, the vagus nerve is a part of a reflex that prevents or neutralizes excessive inflammation in response to tissue injury and infection. Sepsis is a prototypical condition in which an early unrestrained production of cytokines initiates a systemic response involving chemokines, amines, and activation of the complement and of the coagulatory systems eventually leading to disrupted vascular integrity, hypotension, and shock. HMGB1 is a recognized player in the late phases of sepsis. Pioneering studies have shown that stimulation of the vagus nerve or administration of cholinergic agents or selective agonists of the alpha7 nicotinic acetylcholine receptor abate HMGB1 systemic levels and improve animal survival in endotoxaemia or upon cecal ligation and puncture, a standardized model of septic peritonitis. Activation of the alpha7 nicotinic acetylcholine receptors represents a key event in the anti-inflammatory reflex, since it could be responsible for NF-κB nuclear translocation inhibition and thus for the restoration of homeostasis *via* suppression of pro-inflammatory cytokines generation and release (150, 151).

Of importance, this homeostatic system is activated even in sterile conditions, limiting the tissue damaging actions of NF-κB activation and HMGB1 expression in response to heart or hepatic ischemia–reperfusion injury (152, 153). The involvement of this pathway in systemic vasculitis has not been studied extensively so far. However, the accumulating evidence on the role that HMGB1 plays in persisting vascular inflammation (see above) and the possibility to pharmacologically exploit its anti-inflammatory actions (154) suggest that these studies might underpin the development of novel and effective therapeutic strategies.

## Concluding Remarks

High mobility group box 1 is the best characterized alarmin, and its recognition plays a role that is more and more appreciated in several apparently unrelated conditions, in which inflammation does not abate with the original noxa but *per se* causes self-sustaining cell and tissue damage. Many factors contribute to make systemic vasculitis a particularly attractive scenario to dissect the complex biology of HMGB1. These include the characteristics of the pathogenesis of vasculitis, which stem from a deregulated interaction between leukocytes, endothelial cells, and vessel wall cells, and the increasing understanding of the mechanisms that physiologically regulate the homeostatic response of vessels to injury. Increasing knowledge of the immunobiology of HMGB1 will form a foundation for novel targeted immune strategies aimed at specifically targeting the early events in the natural history of vasculitis.

## Author Contributions

NM, PR-Q, and AM selected the bibliography and wrote the manuscript.

## Conflict of Interest Statement

The authors declare that the research was conducted in the absence of any commercial or financial relationships that could be construed as a potential conflict of interest.
